# Personal

**Published:** 1908-04

**Authors:** 


### PERSONAL.

Dr. Harry Radley Trick, of Buffalo, has been appointed by Col. George C. Fox, assistant surgeon of the 74th regiment, holding the rank of captain.

---

Dr. Lawrence H. Smith, a graduate of the University of Buffalo, class of 1907, and who has since been serving as interne at the Buffalo General Hospital, will locate at East Aurora N. Y., early in April, for the practice of his profession.

